# Kinder- und jugendpsychiatrische Versorgung 2019 in Österreich – Stufen der Versorgung, Ist-Stand und Ausblick

**DOI:** 10.1007/s40211-020-00374-6

**Published:** 2020-11-30

**Authors:** Rainer Fliedl, Berenike Ecker, A. Karwautz

**Affiliations:** 1 Lindauergasse 27/2, 1160 Wien, Österreich; 2 ÖGKJP, Wien, Österreich; 3 Universitätsklinik für Kinder- und Jugendpsychiatrie, General Hospital of Vienna, Medizinische Universität Wien, Währinger Gürtel 18–20, 1090 Wien, Österreich

**Keywords:** Versorgung, Österreich, Kinder- und Jugendpsychiatrie, Care, Austria, Child and Adolescent Psychiatry

## Abstract

**Hintergrund:**

Kinder- und jugendpsychiatrische Störungen sind häufig, die Versorgung in Österreich ist erst im Aufbau.

**Methoden:**

Wir untersuchten, ob sich die fachärztliche Versorgungslage seit Etablierung des Sonderfaches verbessert hat.

**Ergebnisse:**

Es besteht weiter eine große Heterogenität der Versorgungslage zwischen den Bundesländern, Die Mangelfachregelung in der derzeitigen Form ist nicht ausreichend, um die Ausbildung im Sonderfach zu konsolidieren und eine Vollversorgung in absehbarer Zeit zu erreichen.

**Conclusion:**

Es werden Anregungen erarbeitet, wie die Versorgungssituation in der Zukunft verbessert werden könnte.

## Summary

**Background:**

Child and adolescent psychiatric disorders are prevalent, their care in Austria is still in progress.

**Methods:**

We investigated whether the child and adolescent psychiatric care made progress dsince the establishment of the medical special discipline.

**Results:**

There remains massive heterogeneity between the Austrian provinces. The “Mangelfachregelung” in ist current version is insufficient in order to consolidate our medical special discipline and to guarantee full carewithin the next years.

**Conclusion:**

Hints are given how the situation of care in our discipline can be strengthened.

## Einleitung

Durch die Österreichische Gesellschaft für Kinder- und Jungendpsychiatrie, Psychosomatik und Psychotherapie (ÖGKJP) werden seit vielen Jahren die landesweiten Versorgungsdaten erhoben und ausgewertet [1–3]. Auf Basis der gewonnenen Ergebnisse werden Empfehlungen für die Weiterentwicklung der kinder- und jugendpsychiatrischen Versorgung (KJP-Versorgung) in Österreich abgeleitet. Die vorliegende Arbeit beinhaltet Daten aus den Jahren 2012 bis 2019. Die Daten für 2019 (mit Stichtag 31.12.2019) wurden im Frühjahr 2020 bei den Vorständen der jeweiligen Abteilungen erhoben.

In der vorliegenden Arbeit folgt zuerst ein Überblick zum Versorgungskonzept und den Stufen der Versorgung. In weiterer Folge werden die Bereiche stationäre Versorgung, ambulante Versorgung und Versorgung durch den niedergelassenen Bereich entlang der erhaltenen Daten beschrieben, die Ausbildungssituation diskutiert und auf innovative und kooperative Aspekte im Versorgungskontext hingewiesen.

## Epidemiologie psychischer Störungen in Österreich und quantitative Annäherung an die notwendigen Behandlungsplätze

In der für Österreich repräsentativen Erhebung zu psychischen Störungen gemäß DSM‑5 bei 10–18-jährigen Jugendlichen, der MHAT-Studie („Mental Health in Austrian Teenagers“) fanden wir [4] eine Halbjahresprävalenz von 23,9 % psychischer Störungen sowie eine Lebenszeitprävalenz von 35,8 %. Fast jede(r) vierte Jugendliche leidet aktuell unter einer psychischen Störung. Per definitionem bedarf jede psychische Krankheit einer Behandlung, allerdings nicht jede einer akuten und unmittelbaren Intervention. Angelehnt an die Kriterien von Packard [5] (Guidelines to use of axis V) besteht bei einem GAF („Global Assessment of Functioning“) von größer/gleich 70 Behandlungsbedürftigkeit, bei GAF Werten unter 70 akute Behandlungsbedürftigkeit. Wir konnten für die Gesamtstichprobe für 8 % der österreichischen jugendlichen Population (*n* = 780.185; 10–18 Jahre; Statistik Austria 2014) eine DSM‑5 Diagnose und einen GAF von über 70 feststellen, für 14 % bei Vorliegen einer Diagnose einen GAF von kleiner als 70. 78 % der Jugendlichen waren seelisch gesund.

Das bedeutet in Zahlen: 170.800 Jugendliche in Österreich leiden 2014 an einer psychischen Störung, davon sind 106.800 akut behandlungsbedürftig. Pro 1000 Jugendliche sind das 171 Menschen (20 pro 1000 Einwohner*innen Österreich) mit einer psychischen Krankheit, 107 davon (12,5 von 1000 Einwohner*innen in Österreich) wären akut behandlungsbedürftig.

Da durchschnittlich über alle Diagnosen knapp die Hälfte der in der MHAT-Studie identifizierten Jugendlichen mit einer Erkrankung bisher nirgends wegen dieser vorstellig wurden und keine adäquate Behandlung aufgesucht haben besteht hier eine hohe Dunkelziffer zu behandelnder Betroffener.

## Versorgungsprinzipien, Versorgungskonzept und Stufen der Versorgung

Kinder- und jugendpsychiatrische und -psychotherapeutische Versorgung (KJPP-Versorgung) folgt dem Prinzip der Gemeindenähe und der Grundregel ambulant vor stationär [6]. Um dieses gestufte Versorgungsmodell wirksam aufzubauen, bedarf es einer definierten und differenzierten Beschreibung der einzelnen Bausteine der Versorgung und regional wirksamer Modelle der Kooperation. Um diese Kooperation zu ermöglichen, ist es notwendig, die einzelnen Bausteine quantitativ und qualitativ mit den dafür notwendigen Ressourcen auszustatten.

Eine wirksame und effiziente Gestaltung dieses gestuften Versorgungsmodells samt Berechnungsschlüssel [6] findet sich in Tab. 1. Es bedarf zudem regional wirksamer Modelle der Kooperation der mit der Behandlung und Betreuung von Kindern und Jugendlichen verantworteten Institutionen.

**Table 1:** 

	Bausteine der Versorgung	Berechnungsschlüssel
Stationär	Intensivbehandlung *Behandlungsform „I“ im LKF-System*	0,1 pro 1000 Einwohner*innen
	Behandlungsplätze *Behandlungsform „A“ im LKF-System*	
	Eltern-Kind-Plätze *Behandlungsform „E“ im LKF System*	
	Tagesklinische Behandlung *Behandlungsform „T“ im LKF-System*	
	Mobile Plätze Home-Treatment integrative Versorgung *Fehlen im LKF-System*	
Ambulant	Ambulanzen oder Ambulatorien	Pro 250.000 Einwohner*innen
	Niedergelassene Fachärzt*innen	Pro 80.000 auf Einwohner*innen
Komplementärbereich	Beratungsstelle mit KJPP-Kompetenz für Kinder und Jugendliche und ihre Familien	Pro 80.000 Einwohner*innen
	Ambulante und stationäre Einrichtungen der Jugendhilfe	–
	Einrichtungen im Bildungssystem und des Arbeitsmarktservices (AMS) wie Beratungslehrer*innen, Schulsozialarbeit, Sonderkindergärtner*innen	–
Partizipation	Betroffenen- und Elternvertretungen	–

*LKF* Leistungsorientierte Krankenhausfinanzierung

Im Kernbereich der KJPP-Versorgung sind im stationären Bereich psychosomatische und sozialpädiatrische Plätze und im ambulanten Bereich neuropädiatrische und psychotherapeutische Ambulanzen abzugrenzen und kooperativ weiter zu entwickeln. Auch die anderen Einrichtungen im Komplementärbereich im Rahmen der Jugendhilfe, der Schule und anderer Institutionen, die Kindern und Jugendlichen zur Verfügung gestellt werden, werden nicht im Detail beschrieben. Es bedarf aber einer gemeinsamen Planung und Weiterentwicklung um optimale Synergien in den überlappenden Aufgabenbereichen zu erzielen.

Der Berechnungsschlüssel der Bausteine bezieht sich auf den Bevölkerungsstand des Jahres 2019, dieser verändert sich kontinuierlich. 2019 hatte Österreich 8.901.064 (Statistik Austria 2020) [7] Einwohner*innen (Ew.) mit einem Anteil von 19,3 % Minderjährigen, das entspricht einem Bevölkerungswachstum von 0,5 % im Jahr 2019 [12]. Für das Jahr 2030 ist die Bevölkerungsprognose bei 9.210.146 Ew. (Statistik Austria 2020 [13]), der Anteil an Minderjährigen von 19,3 % wird gleichbleiben. Dies entspricht einem Wachstum von 5 %. In dieser Arbeit wird die IST–SOLL Divergenz von 2019 angesprochen, sinnvolle Planungszahlen liegen in einzelnen Regionen bis zu 10 % über diesen Werten.

Diese spezifischen Versorgungsangebote und -einrichtungen sind andernorts [8–10] bereits ausführlich beschrieben und über Qualitätsstandards definiert.

Aus den Daten der MHAT-Studie ist ersichtlich, dass in einer Standardversorgungsregion von 500.000 Einwohner*innen (Ew.) 6250 Kinder und Jugendliche akut behandlungsbedürftig (GAF unter 70) sind.

Quantitativ lässt sich der Bedarf in einer Standardversorgungsregion wie folgt zusammenfassen:

- Eine stationäre Einrichtung für KJPP mit 50 Behandlungsplätzen (0,1‰),
- Zwei kinder- und jugendpsychiatrische Ambulanzen bzw. Ambulatorien,
- Sechs bis sieben niedergelassene Fachärzt*innen für Kinder- und Jugendpsychiatrie,
- Sechs bis sieben Miniambulatorien/Beratungsstellen mit kinder- und jugendpsychiatrischer Kernkompetenz [11].

Bezogen auf das Bundesgebiet Ö ergibt sich der in Tab. 2 dargelegte Bedarf.

**Table 2:** 

Einrichtungen	Berechnungsschlüssel	Bedarf
Stationäre und tagesklinische Plätze (BMZ)	0,1 pro 1000 Ew	890
Ambulanzen oder Ambulatorien	1 pro 250.000 Ew	36
Niedergelassene Fachärzt*innen	1 pro 80.000 auf Ew	111

*BMZ* Bettenmessziffer

## Versorgungslage in Österreich

### Stationärer Bereich

2019 hatte Österreich 8.901.064 Einwohner*innen [14]. Die Mitversorgung von bayerischen Patient*innen durch Salzburg und von Patient*innen aus Liechtenstein durch Vorarlberg wird nicht eingerechnet.

Zur stationären kinder- und jugendpsychiatrischen Versorgung stehen zur Zeit 12 Krankenhausabteilungen zur Verfügung. Von diesen Abteilungen haben sechs dislozierte Außenstellen mit einer Ambulanz und fünf dislozierte Außenstellen mit tagesklinischen Plätzen. Weiters gibt es die Ambulanz und Tagesklinik des Psychosozialen Dienstes (PSD) Wien.

Die Klinik Floridsdorf wird, da sie im Aufbau begriffen ist und mit 31.12.2019 nicht eigenständig versorgungswirksam ist, im Verbund mit dem der Abteilung für Kinder- und Jugendpsychiatrie und Psychotherapie (KJPP) der Klinik Hietzing beschrieben.

Die Versorgungsregionen haben eine Größe zwischen 397.139 Einwohner*innen (Vorarlberg) und 1.490.279 Einwohner*innen (Oberösterreich) (siehe auch Karte 1). Eine Standardversorgungsregion wird mit 500.000 Einwohner*innen gerechnet [11].

Besonders in flächenmäßig großen Versorgungsregionen wie beispielsweise Oberösterreich und Steiermark wären weitere Standorte notwendig, um eine Regionalisierung der Versorgung zu erzielen.

**Figure 1:**
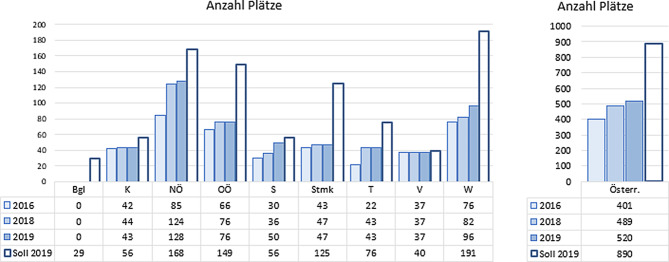

Seit 2016 kam es österreichweit zu einem Zuwachs von 119 Behandlungsplätzen. Davon waren 38 Plätze Stationär und 81 Tagesklinisch. Siehe auch Abb. 1. Dieser Zuwachs wurde durch folgende Erweiterungen erzielt.

- Niederösterreich: Die Eröffnung der Außenstellen der Krankenhausabteilungen Mistelbach, Wiener Neustadt und Waidhofen an der Thaya und den Neubau der KJPP im Landeskrankenhaus Mauer.
- Salzburg: Der Neubau der Uniklinik, die Eröffnung der KJPP im Krankenhaus Schwarzach
- Steiermark: Die tagesklinischen Plätze im Krankenhaus Leoben,
- Tirol: Der Neubau der KJPP im Landeskrankenhaus Hall und die Tagesklinik in Innsbruck.
- Wien: Die Plätze für die tagesklinische Behandlung an der Klinik Floridsdorf und eine Erhöhung der Plätze an der Klinik Hietzing.

**Figure 2:**
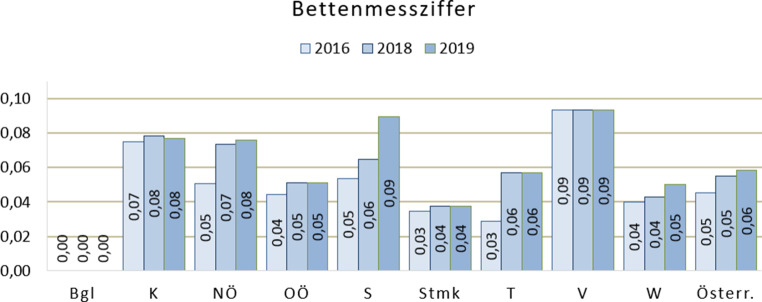

Mit dem Zuwachs der Plätze hat sich die Bettenmessziffer (BMZ) in den letzten drei Jahren von 0,05auf 0,06 erhöht. Unter der BMZ versteht man die Zahl der Behandlungsplätze, die pro 1000 Einwohner benötigt wird, um eine ausreichende Versorgung zu gewährleisten. Der Wert von 0,1 wurde in Salzburg und Vorarlberg nahezu erreicht. Die niedrigsten Bettenmessziffern finden sich in der Steiermark und Wien, im Burgenland besteht keine eigene stationäre Versorgung, es besteht aber eine Mitversorgung durch Niederösterreich und die Steiermark und wird dort mitberechnet (Abb. 2).

### Tageskliniken

**Figure 3:**
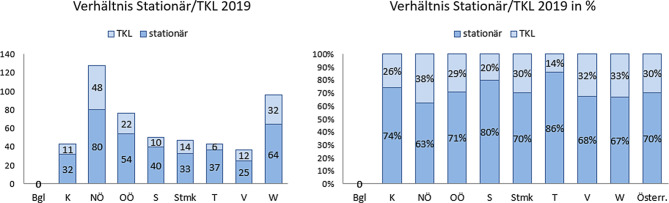

Das Verhältnis von tagesklinischen zu vollstationären Plätzen fällt im Jahr 2019 österreichweit mit einem Verhältnis von 30 % zu 70 % relativ homogen aus. Im Vergleich zu 2018, wo das Verhältnis bei 27 % zu 73 % lag, hat der Anteil an tagesklinischen Betreuungsmöglichkeiten leicht zugenommen. Tirol stellt eine Ausnahme dar, da es dort bis auf die Ambulanz in Innsbruck noch keine dislozierten Einrichtungen gibt. Siehe auch Abb. 3.

### Home-Treatment und Eltern-Kind-Plätze

Eltern-Kind-Aufnahmen sind in einigen Abteilungen (z. B. LKH Hall, LKH Mauer) möglich, in der Gesamtheit der Versorgung aber noch keine verlässliche Säule.

Für den Bereich des Home-Treatments wurden Konzepte erstellt. Vorbilder dafür gibt es z. B. in der Kinder- und Jugendpsychiatrie Hamburg für Psychosen und in Aachen für Essstörungen. Es bedarf aber sowohl einer Verankerung im LKF-System als auch einer praktischen Umsetzung in den Einrichtungen.

### Stationäre kinder- und jugendpsychiatrische Versorgungsregionen

**Figure 4:**
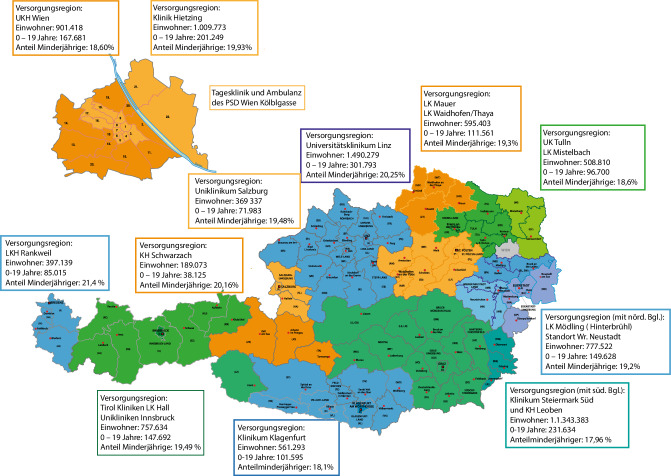

Da die Versorgungsregionen nicht an die Bundesländergrenzen orientiert sind, ist eine Darstellung (siehe Abb. 4) der KJPP-Versorgungsregionen sinnvoll. Einige Bundesländer haben sinnvollerweise mehrere Versorgungsregionen. Patient*innen vom Nord- und Mittelburgenland werden von der KJPP Hinterbrühl mitversorgt, jene vom südlichen Burgenland von der KJPP in Graz. Gewissermaßen vergleichbar stellt sich die Situation in Osttirol, das zum Teil von Kärnten mitversorgt wird. Zwischen den einzelnen Bundesländern ist daher zu klären, welche Landesregierung die Verantwortung für die Versorgung dieser Regionen übernimmt und vorantreibt.

Die stationäre und tagesklinische Versorgung auf der Ebene der Versorgungsregionen wird in Tab. 3 dargestellt. Neben der Einwohnerzahl und dem Anteil der Minderjährigen werden der Soll-stand mit dem Ist-stand verglichen und in der jeweils erreichten Bettenmessziffer als wesentlichster Bezugsgröße zusammengefasst.

**Table 3:** 

Versorgungsregionen	Einwohner	Anzahl stationär	Anzahl TKL	Summe Stat. + TKL	0–19	% Minderjährige	Soll Plätze	Fehlende Plätze	Bettenmessziffer
Klinikum Klagenfurt/W	561.293	32	11	43	101.595	18,10	56	13	0,077
LK Mauer	595.403	30	16	46	118.014	19,82	60	14	0,077
LK Mödling (Hinterbrühl)	777.522	30	16	46	149.628	19,24	78	32	0,059
UK Tulln	508.810	20	16	36	96.700	19,01	51	15	0,071
UK Linz	1.490.279	54	22	76	301.793	20,25	149	73	0,051
Klinikum Salzburg	369.337	30	10	40	71.983	19,49	37	−3	0,108
Krankenhaus Schwarzach	189.073	10	0	10	38.125	20,16	19	9	0,053
Stmk Standort Süd	1.343.383	33	14	47	241.440	17,97	134	87	0,035
LKH Hall	757.634	37	6	43	147.692	19,49	76	33	0,057
LKH Rankweil	397.139	25	12	37	167.681	21,41	40	3	0,093
UK Wien	901.418	28	8	36	167.681	18,60	90	54	0,040
Klinik Hietzing und Klinik Floridsdorf und PSD Kölblgasse^a^	1.009.773	36	24	60	201.249	19,93	101	65	0,059

^a^Die 12 TKL Plätze des KH Floridsdorf und die 12 Plätze der Tagesklinik des PSD in der Kölblgasse haben keine eigenständige Versorgungsregion, sie wurden der Klinik Hietzing zugeordnet, damit diese Plätze in der Tabelle aufscheinen

### Ambulante Versorgung

Wie in den Versorgungsprinzipien angesprochen, stellen sowohl Ambulanzen und Ambulatorien, als auch Niedergelassene wichtige Elemente der Versorgung dar, da über diese unterschiedlichen Angebote verschiedene Zugänge zur kinder- und jugendpsychiatrischen Versorgung geschaffen werden und damit eine höhere Versorgungsprävalenz erreicht werden kann.

### Ambulatorien und Ambulanzen

Grundsätzlich ist eine ambulante Einrichtung für 250.000 Einwohner*innen notwendig. Dies entspricht für Gesamtösterreich 36 Ambulanzen oder Ambulatorien. Ein exakter Vergleich der Ambulanzen ist nicht möglich, da die zur Verfügung stehenden Personalressourcen der einzelnen Ambulanzen und damit ihre Versorgungskapazität unterschiedlich sind.

Zum Stichtag am 31.12.2019 gab es 22 ambulante Einrichtungen in Österreich. Hier werden die Boje und Extended Soulspace nicht eingerechnet, da sie spezifische Aufgaben und Aufträge haben. Diese Einrichtungen werden bei *Spezialambulanzen *beschrieben.

Wie aus Tab. 4 hervorgeht, besteht im Burgenland und in Salzburg eine gute Ausstattung mit Ambulanzen und/oder Ambulatorien. Der eklatanteste Mangel besteht in Wien, Oberösterreich und in der Steiermark.

**Table 4:** 

	Soll	Ist
Burgenland	1	2
Kärnten	2	1
Niederösterreich	7	5
Oberösterreich	6	1
Salzburg	2	3
Steiermark	5	2
Tirol	3	2
Vorarlberg	2	2
Wien	8	4
Österreich	36	22

### Spezialisierte Angebote an den Ambulanzen und Ambulatorien

Trotz des erheblichen Mangels an der Zahl ambulanter Einrichtungen wurde eine spezifische Versorgung für unterschiedliche Patient*innengruppen aufgebaut, in denen, den Ressourcen entsprechend, spezifische Behandlungsangebote erteilt werden können. Viele dieser Angebote sind an mehreren Abteilungen möglich, ein Schwerpunkt liegt an den Universitätskliniken (alphabetisch).

- ADHS
- Autismus
- Drogen
- EEG
- Entwicklung
- Essstörungen
- Forensik
- Genderinkongruenz
- Geschlechtsdysphorie
- Kinder psychiatrisch kranker Eltern
- Kinder suchtkranker Eltern
- Psychologische Diagnostik
- Psychose-Nachsorge Psychotherapie
- Psycho-Diabetologie
- Säugling/Kleinkind
- Schmerz
- Tourette Syndrom
- Transkulturelle Probleme
- Traumafolgestörungen
- UMF (unbegleitete minderjährige Flüchtlinge)

### „Spezialambulanzen“

- *Ambulatorium die Boje*
  Die Boje hat einen spezifischen Versorgungsauftrag in der interdisziplinären Versorgung akut traumatisierter Kinder und Jugendlicher. Die Versorgungsregion ist Wien, Niederösterreich und das Burgenland. Die Ausstattung der Einrichtung entspricht den Standards eines Ambulatoriums [10] Durch den niederschwelligen Zugang und der Möglichkeit zu Akutinterventionen können Kinder und Jugendliche in traumatisierenden Situationen in kurzer Zeit Hilfe bekommen.
- *Kinder- und Jugendpsychiatrische Ambulatorium mit Tagesklinik – Extended Soulspace des PSD Wien*
  Das Ambulatorium hat mit der Versorgung von Kindern und Jugendlichen im Rahmen von Maßnahmen der Jugendhilfe einen spezifischen Versorgungsauftrag. Neben der ambulanten Behandlung von Kindern und Jugendlichen werden auch Hausbesuche in Wohngemeinschaften gemacht, weiters stehen 16 Plätze für tagesklinische Behandlung zur Verfügung. Die Einrichtung ist ein wichtiges Behandlungsangebot für ein besonders gefährdetes Klientel.

### Nebentätigkeiten der Ambulanzen

Neben der primären ambulanten Versorgungstätigkeit werden Konsiliardienste für andere Abteilungen (Erwachsenenpsychiatrie, Pädiatrie, Drogenstation, Kinderchirurgie, Jugendforensik, Onkologie, Transplant, Intensiv, Neonatologie), Konsiliar- und Liaisondienste für Krisenzentren, Wohngemeinschaften und Heime der Jugendhilfe, und KJPP-Versorgung der unbegleiteten minderjährigen Flüchtlinge angeboten. Für diese Leistungen stehen nur an wenigen Abteilungen gesonderte Personalressourcen zu Verfügung. Gleichzeitig sind diese Aufgaben von großer Relevanz, da dadurch die die psychiatrische Versorgung in diesen Bereichen verbessert werden kann.

### Niedergelassene Kassenärzt*innen

**Figure 5:**
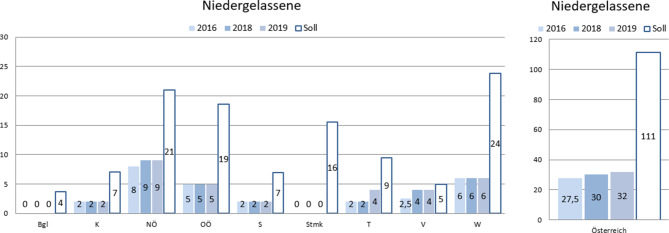

Seit 2016 kam es österreichweit zu einem Zuwachs von 4,5 Kassenplätzen. Damit wurden 36 % der für die Versorgung notwendigen Kassenplätze geschaffen. Besonders bedauerlich ist, dass es im Burgenland und in der Steiermark keine Kassenordinationen gibt und dies auch eine strukturelle Schlechterstellung der Patient*innen in diesen Bundesländern zur Folge hat. Siehe auch Abb. 5.

## Die Fachärzt*innenausbildung im Fach Kinder- und Jugendpsychiatrie und Psychotherapeutische Medizin

**Figure 6:**
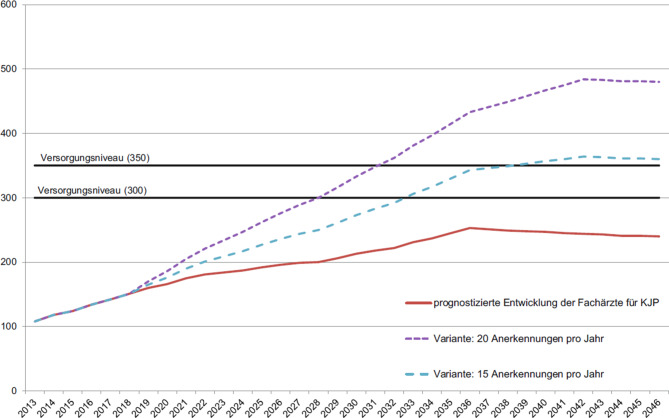

Die problematische Situation des Mangels an Fachärzt*innen wurde von Hartl und Karwautz [2] differenziert beschrieben. In dieser Arbeit wird ausgehend von 2013 verdeutlicht, dass es unter günstigen Bedingungen mehr als 10 Jahre dauern wird, um die erforderliche Anzahl an Fachärzt*innen auszubilden (siehe auch Abb. 6).

Es absolvierten in den Jahren 2015 bis 2019 zwischen neun und fünfzehn Ärzt*innen die Facharztprüfung, damit konnte bei einem Durchschnitt von 12 die Untergrenze der Prognose nicht erreicht werden. Die Entwicklung der letzten Jahre lässt eine günstige Prognose (20 oder 15 Anerkennungen pro Jahr) bis jetzt daher nicht erwarten, das Fach stagniert also zahlenmäßig und das Erreichen der Vollversorgung ist jetzt absehbar (Stand 2019/20) weiter nicht in Aussicht.

Es bleibt daher wichtig, eine größere Anzahl an Assistenzärzt*innen auszubilden, um dem Bedarf an kinder- und jugendpsychiatrischer Versorgung quantitativ Rechnung zu tragen und auch die damit in Verbindung stehende qualitative Weiterentwicklung des Faches voranbringen zu können.

Mit dem Stichtag 31.12.2019 gab es an den Befragten Einrichtungen 96 genehmigte Ausbildungsstellen. Diese geringe Anzahl kann auch durch die Mangelfachregelung in der ÄAÖ 2015 §37 (1) die bis 31.05.2021 gültig ist, nicht deutlich verbessert werden. Eine Verbesserung wäre mit der Regelung „Eine Fachärzt*in kann zwei Assistent*innen ausbilden“ möglich.

Wegen des Mangels an Fachärzt*innen konnten österreichweit von den 125 Dienstposten für Fachärzt*innen 25 Dienstposten nicht besetzt werden. Da diese Facharztstellen auch in den nächsten Jahren nur sehr langsam besetzbar sind, wird es zu keiner Vermehrung der Ausbildungsplätze kommen.

Wir können daher mit etwa 19 neuen Fachärzt*innen pro Jahr rechnen (entsprechend der Anzahl der Absolventen der Fachärzt*innenprüfung). Da circa 1/3 der neuen Fachärzt*innen nicht im öffentlichen Gesundheitsbereich arbeiten wird, sondern in privaten Ordinationen (welche üblicherweise nicht voll versorgungsrelevant sind), können wir zur Zeit nur mit einem Zuwachs von 12–13 Fachärzt*innen rechnen, die in komplett versorgungsrelevanten Strukturen tätig werden – diese Anzahl wird in den nächsten Jahren gerade die Anzahl an Fachärzt*innen, die in Pension gehen, decken.

Zum Stichtag waren 4 Ausbildungsstellen nicht besetzt. Dies ist nachvollziehbar, da zu diesem Zeitpunkt an zwei Abteilungen die Ausbildungsstellen nicht durch Fachärzt*innen abgedeckt wurden und daher nicht besetzbar waren und an einer Abteilung keine Dienstposten zur Verfügung standen um Assistenzärzt*innen anzustellen.

Eine Vermehrung der Ausbildungsstellen ist daher primär nur durch eine Veränderung der Mangelfachregelung möglich, da in den nächsten Jahren nur mit einem sehr langsamen Zuwachs an Fachärzt*innen an den Abteilungen gerechnet werden kann.

Die meisten Bewerbungen für eine Assistentenstelle gibt es an den Universitätskliniken, an denen im Rahmen des Medizinstudiums Kinder- und Jugendpsychiatrie in ausreichender Stundenanzahl unterrichtet wird und auch die Möglichkeit des Klinisch-Praktischen Jahres gern in Anspruch genommen wird. Die weitere Integration des Faches in die universitäre Ausbildung ist damit eine wichtige Maßnahme, um den Studierenden das Fach bekannt zu machen und ihnen zu ermöglichen, sich dafür zu interessieren. Auch das Instrument des Status von Krankenanstalten als Lehrkrankenhäuser wäre zielführend, um zukünftigen Kollegen die aus den Bundesländern kommen, die regional nahen Abteilungen nahezubringen und für Ausbildung in KJPP zu gewinnen.

## Interdisziplinarität in der Kinder- und Jugendpsychiatrie

**Figure 7:**
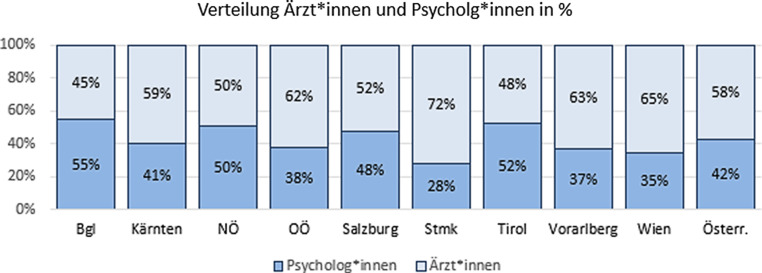

Kinder- und jugendpsychiatrische Arbeit erfordert ein interdisziplinäres therapeutisches Team. Dazu bedarf es unterschiedlicher Berufsgruppen. Ein wichtiges Gegenüber sind die klinischen Psycholog*innen. Es sind insgesamt 130 Psycholog*innen (VZÄ) in den ambulanten und stationären kinder- und jugendpsychiatrischen Einrichtungen beschäftigt. In den meisten Abteilungen sind Psycholog*innen mit Fallführungsaufgaben betraut. Bei den stationären Einrichtungen sind meist mehr Ärzt*innen als bei den ambulanten Einrichtungen tätig, da von den Ärzt*innen die Nachtdienste geleitet werden. Siehe auch Abb. 7.

## Kooperation, Transition und Nachhaltigkeit der Behandlung

Die kinder- und jugendpsychiatrische Versorgung erfolgt in einer engen Kooperation und Vernetzung mit anderen medizinischen Fächern (Kinderheilkunde, Psychiatrie) und den nicht-medizinischen Säulen der Jugendhilfe, Sozialhilfe und den edukativen Bereichen (Kindergarten, Schule und Arbeitsmarktförderungen). Kooperationen und Übergänge in der Behandlung sind damit relevante Faktoren, sowohl für die Schaffung und den Erhalt des Zuganges zu einer kinder- und jugendpsychiatrischen Behandlung (Versorgungsprävalenz), als auch für die Erlangung einer nachhaltigen Wirksamkeit der Behandlungen.

In den Bundesländern wurden unterschiedliche Kooperationsprojekte mit Institutionen umgesetzt, die schwerpunktmäßig mit Kindern und Jugendlichen arbeiten, wie zum Beispiel die Kindernetzwerke. Es sind zudem spezifische Kooperationen zwischen Jugendhilfe und KJPP entstanden und verschiedene Ansätze der Kooperation zwischen Schule und KJPP.

## Vollversorgung als Ziel

**Figure 8:**
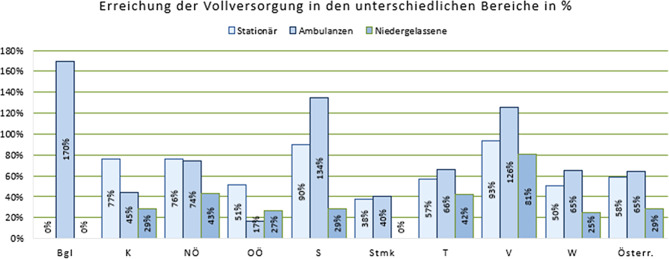

Ziel ist die bundesweite Vollversorgung im Sonderfach Kinder- und Jugendpsychiatrie und Psychotherapeutische Medizin. Abb. 8.

## Fazit

Es besteht die Dynamik eines sich in Entwicklung befindlichen Sonderfaches der Medizin.

13 Jahre nach Gründung des Sonderfaches besteht weiterhin große Heterogenität der Versorgungslage zwischen den Bundesländern, sowohl auf der stationären als auch tagesklinischen und ambulanten/niedergelassenen Ebene (Extrembeispiel „Steiermark, Wien“ vs. „Salzburg, Vorarlberg“). Um die Versorgung zu verbessern, muss die Sonderfachausbildung vorangetrieben werden, damit eine ausreichende Anzahl an Ärzt*innen ausgebildet wird. Die Mangelfachregelung in der derzeitigen Form ist nicht ausreichend, um die Ausbildung im Sonderfach zu konsolidieren und eine Vollversorgung in absehbarer Zeit zu erreichen. Dazu bedarf es dringend einer Bewegung von Seiten der Österreichischen Ärztekammer.

In die Leistungsorientierte Krankenhausfinanzierung LKF müssen auch mobile Angebote integriert werden, damit in Österreich moderne Behandlungsstrukturen geschaffen werden können, wie sie in Deutschland und der Schweiz zum Standard gehören.
